# The Physiological Regulation of Skeletal Muscle Fatty Acid Supply and Oxidation During Moderate-Intensity Exercise

**DOI:** 10.1007/s40279-015-0394-8

**Published:** 2015-11-09

**Authors:** Gerrit van Hall

**Affiliations:** Clinical Metabolomics Core Facility, Department of Biomedical Sciences, Faculty of Health and Medical Sciences, Rigshospitalet, University of Copenhagen, Section 7652, 9 Blegdamsvej, 2100 Copenhagen, Denmark

## Abstract

Energy substrates that are important to the working muscle at moderate intensities are the non-esterified fatty acids (NEFAs) taken up from the circulation and NEFAs originating from lipolysis of the intramuscular triacylglycerol (IMTAG). Moreover, NEFA from lipolysis via lipoprotein lipase (LPL) in the muscle of the very-low-density lipoproteins and in the (semi) post-prandial state chylomicrons may also contribute. In this review, the NEFA fluxes and oxidation by skeletal muscle during prolonged moderate-intensity exercise are described in terms of the integration of physiological systems. Steps involved in the regulation of the active muscle NEFA uptake include (1) increased energy demand; (2) delivery of NEFA to the muscle; (3) transport of NEFA into the muscle by NEFA transporters; and (4) activation of the NEFAs and either oxidation or re-esterification into IMTAG. The increased metabolic demand of the exercising muscle is the main driving force for all physiological regulatory processes. It elicits functional hyperemia, increasing the recruitment of capillaries and muscle blood flow resulting in increased NEFA delivery and accessibility to NEFA transporters and LPL. It also releases epinephrine that augments adipose tissue NEFA release and thereby NEFA delivery to the active muscle. Moreover, NEFA transporters translocate to the plasma membrane, further increasing the NEFA uptake. The majority of the NEFAs taken up by the active muscle is oxidized and a minor portion is re-esterified to IMTAG. Net IMTAG lipolysis occurs; however, the IMTAG contribution to total fat oxidation is rather limited compared to plasma-derived NEFA oxidation, suggesting a complex role and regulation of IMTAG utilization.

## Introduction

Limitation of carbohydrate and lipid transfer from the microvascular system to the muscle cell occurs at the onset of exercise when the delivery and transport systems are not optimal and during continuous exercise above moderate intensities of 50 % of maximal pulmonary oxygen uptake ( VO_2max_). At higher continuous workloads the extracellular substrate provision rate is not high enough and intracellular stored substrates must also be used, such as glycogen and intramuscular triacylglycerol (IMTAG) [1]. Due to complexity in the regulation of fat metabolism, it is still unknown what limits active muscle fat oxidation [2–4]. Various suggestions have been put forward, from limitation in the non-esterified fatty acids (NEFAs) delivery to the active muscle, to NEFA uptake into muscle or into the mitochondria or β-oxidation. In addition, the IMTAG not limited by delivery or uptake seems to be not utilized optimally as its pool is not even close to fully utilized [5].

**Fig. 1:**
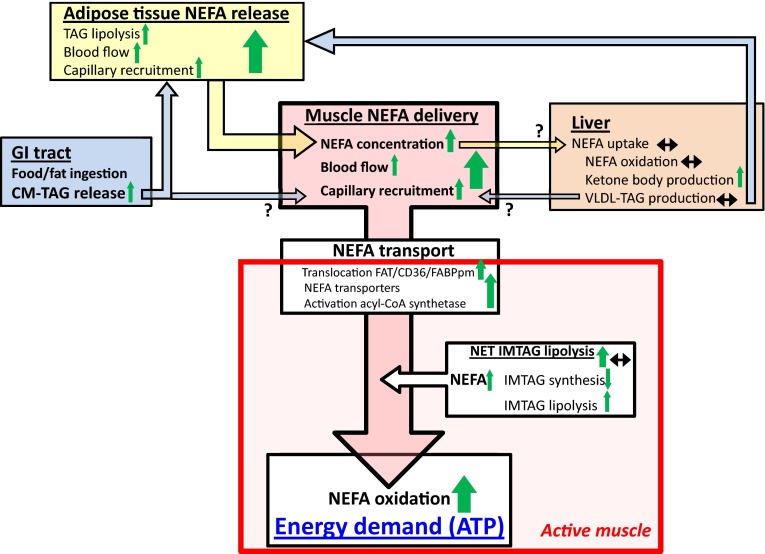
Schematic representation of the control of non-esterified fatty acid (NEFA) fluxes and oxidation during exercise described in terms of the integration of physiological systems. Central in the scheme is the close linear relationship between the active muscle NEFA delivery and uptake/oxidation observed during continuous moderate-intensity exercise. The increase in NEFA delivery is caused by an increase in plasma flow that includes capillary recruitment, whereby a larger portion of the total available NEFA transport machinery becomes accessible. Plasma flow and capillary recruitment do not change much with continuous exercise at the same workload; hence, the increase in muscle NEFA delivery and uptake/oxidation with exercise duration is mainly mediated by the increase in NEFA concentration. The increase in the plasma NEFA concentration is facilitated by an increased release of NEFA from adipose tissue via increased adipose tissue triacylglycerol (TAG) lipolysis, adipose tissue blood flow, and capillary recruitment with epinephrine and possible atrial natriuretic peptide as the key regulators during exercise. Liver NEFA uptake at rest and during exercise is substantial but does not seem to change much with exercise, thus it does not have much effect on the plasma NEFA concentration. Mandatory, but likely not limiting for NEFA oxidation in healthy individuals, is the facilitated NEFA uptake by transporters and binding proteins of which fatty acid translocase (FAT/CD36) and fatty acid bounding protein (FABPpm) translocate from the intracellular depot(s) to the plasma membrane with muscle contraction. NEFA from intramuscular TAG (IMTAG) is used during moderate-intensity exercise. The net degradation of IMTAG is caused by a decrease in IMTAG synthesis and maintained or increased lipolysis. Moreover, NEFA originating from either very-low-density lipoproteins (VLDL-TAG), or chylomicrons (CM-TAG) primarily in the fed state, may contribute to the active skeletal muscle NEFA oxidation. Dietary fat reaches the liver, adipose tissue, and skeletal muscle in the form of CM-TAG that undergoes lipolysis by lipoprotein lipase (LPL) and the resulting NEFAs are taken up and either oxidized or esterified by the active muscle (see also Fig. 2). The contribution of VLDL-TAG- and CM-TAG-derived NEFA to the resting and active muscle energy requirements seems limited. *ATP* adenosine triphosphate, *CoA* coenzyme A, *GI* gastrointestinal

The plasma NEFA concentration plays an important role in the NEFA uptake and subsequent oxidation by the active muscle. If the plasma NEFA concentration falls, the rate of muscle NEFA uptake and subsequent oxidation will decline as well. Conversely, an increase in the plasma NEFA concentration will increase the active muscle NEFA uptake and oxidation [6], similar to blood glucose and its uptake by the active muscle [7]. Adipose tissue release of NEFAs is the primary source of NEFAs by which the plasma NEFA concentration is sustained under post-absorptive conditions or increased during exercise [8]. Thus, the control of adipose tissue triacylglycerol (TAG) lipolysis and subsequent release of the NEFAs into the circulation has an important role in the regulation of muscle NEFA uptake and oxidation during exercise. The liver also plays an important role as it has a high NEFA uptake and, thereby, can affect the plasma NEFA concentration, albeit changes in liver NEFA uptake during exercise are not well-described [9]. Moreover, the NEFA delivery to the active muscles (NEFA concentration × plasma flow) is an even better determinant of the NEFA uptake than the NEFA concentration [10]. The blood flow to the active muscles increases several-fold upon exercise, primarily to increase oxygen supply, but it will also increase NEFA delivery. NEFA uptake from the plasma into the cytosol may occur to some extent via passive diffusion but over the past decades it has been shown that the majority of NEFA uptake occurs via facilitated transport and that muscle contraction induces plasma membrane fatty acid translocase (FAT/CD36) and fatty acid binding protein (FABPpm) translocation from the intracellular depots to the plasma membrane [11]. Therefore, NEFA transport into the muscle cells may be a potential regulatory and limiting step in NEFA utilization by muscle during exercise. Once in the cytosol, the NEFA is activated and can either be oxidized or stored in IMTAG. In this review, the control of human in vivo NEFA fluxes and subsequent oxidation by skeletal muscle during prolonged moderate-intensity exercise are described in terms of the integration of physiological systems (Fig. 1).

## Skeletal Muscle Non-Esterified Fatty Acid (NEFA) Oxidation During Exercise from Plasma-Derived NEFA and Intramuscular Triacylglycerol Lipolysis

The regulation of the active muscle fatty acid uptake and subsequent oxidation is complex and may be differently regulated at low, moderate, and high exercise intensities and durations of exercise. Relatively few studies have directly determined human muscle NEFA handling during exercise, with most of the outstanding studies performed in the 1960–1970s [12–15], and even fewer studies have determined IMTAG involvement [6]. The limited, but remarkably consistent, available quantitative and kinetic information originates from prolonged moderate-intensity exercise. The regulation of the active muscle NEFA uptake can be defined by a four-step process, as depicted in Fig. 1, consisting of (1) an increased energy demand by the contracting muscle; (2) delivery of NEFA to the muscle; (3) transport of NEFA into the muscle by fatty acids transporters; and (4) activation of the fatty acids and either oxidation or re-esterified into intracellular lipids and stored into the IMTAG droplets located next to the mitochondria. A similar concept is generally acknowledged for glucose [7, 16] but differences seem to exist with respect to glycogen versus IMTAG as the intramuscular carbohydrate and fat energy stores, respectively.

**Fig. 2:**
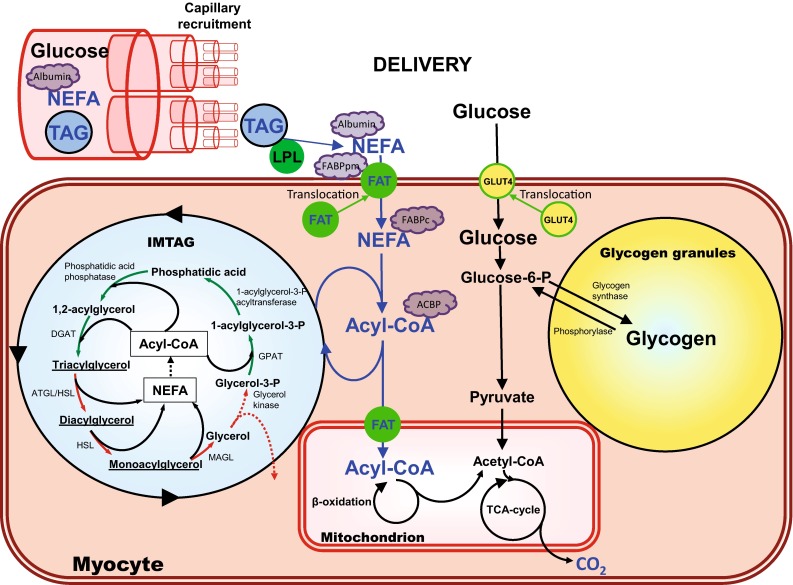
Schematic representation of skeletal muscle energy metabolism. Two pathways in skeletal muscle energy oxidation during exercise can be recognized: the extracellular and intracellular substrate supply. The increase in the extracellular muscle energy supply during exercise is mediated via an increase in the blood substrate delivery of glucose from either carbohydrate intake or liver glycogenolysis and gluconeogenesis, non-esterified fatty acids (NEFA) mainly from adipose tissue, and chylomicron or very-low-density lipoproteins referred to as triacylglycerol (TAG) (see Fig. 1). The increase in delivery of these substrates to the active muscle is mediated by an increase in blood flow, including an increase in capillary recruitment, and substrate concentration. Transport of blood glucose into skeletal muscle is facilitated by glucose transporter-4 (GLUT4) and the long-chain NEFAs via fatty acid transporters (FAT), that also facilitates the transport of NEFA into the mitochondria. The fate of the glucose and NEFA taken up by skeletal muscle is oxidation or storage into glycogen or TAG, respectively. The intracellular energy supply during exercise is immediately increased, mainly via a fast breakdown of glycogen crucial to cover the instantaneously manyfold increase in energy demand going from rest to exercise. The rate of glycogen breakdown decreases with exercise duration, and glucose uptake and subsequent oxidation and later fatty acid oxidation increases. The increase in NEFA availability from intramuscular triacylglycerol (IMTAG) breakdown during exercise is mediated by a reduction in NEFA re-esterification and possibly an increase in IMTAG lipolysis. The role and regulation of the muscle IMTAG turnover rate is unknown. *ACBP* acyl-CoA binding protein, *ATGL* adipose tissue triglyceride lipase, *CoA* coenzyme A, *DGAT* diacylglycerol acyltransferase, *FABP* fatty acid binding protein (*pm* plasma membrane, *c* cytosolic), *FABPm* fatty acid binding protein, *GPAT* glycerol-3-phosphate acyltransferase, *HSL* hormone sensitive lipase, *LPL* lipoprotein lipase, *MAGL* monoacylglycerol lipase, *TCA* tricarboxylic acid

The increased metabolic rate/demand of the active muscle is usually not considered to be a regulatory step. However, it is the main driving force for all physiological regulatory processes. It elicits functional hyperemia, increasing muscle blood flow, the number of perfused capillaries (recruitment), and hormone levels that affect adipose tissue NEFA release, and hence NEFA delivery to the active muscle. Moreover, resting skeletal muscle has very low energy expenditure and thus the demand for energy/adenosine triphosphate is small, implying that an increase in energy demand with exercise causes a surge for substrates that possibly creates a concentration gradient for NEFA between plasma, the interstitial space, cytosol, and entry in the mitochondria (Figs. 1, 2).

The resting muscle blood flow is low and 45–55 % of all NEFA delivered to the muscle is taken up [10, 12, 15, 17] and is clearly dependent on the NEFA concentration [6]. This suggests that the facilitated muscle NEFA uptake is limited/saturable or that the lack of substrate demand and/or re-esterification into intracellular lipids is low, reducing the NEFA gradient for uptake. During moderate-intensity exercise the blood flow to the active muscles increases linearly with the workload [18] and is easily 10- to 15-fold higher during moderate-intensity exercise [17] than at rest, and with the increase in blood flow the blood transit time through the active muscle is substantially reduced [19, 20]. The NEFA fractional extraction decreases to only ~20 % [6, 10, 12–15, 17] as compared to the 45–55 % at rest, despite the massive increase in blood flow and reduced transit time. In addition, the NEFA factional extraction is the same over a wide range of plasma flows [12], i.e., exercise intensities, and is independent of the NEFA concentration over at least a threefold increase in NEFA concentration [6, 15]. Accordingly, the active muscle NEFA uptake is very closely and linearly related to the NEFA delivery, which is the NEFA concentration multiplied by the plasma flow to the active muscle [6, 10, 12–15, 17]. The same relationship is found between NEFA delivery and NEFA oxidation, since 85–100 % of the NEFA taken up by the active muscle is directly oxidized [6, 10, 12–14, 17]. Thus, during exercise the increase in NEFA uptake with delivery is dependent on the functional exercise hyperemia increase in blood flow. However, the linear increase of NEFA delivery and oxidation with the duration of prolonged moderate-intensity exercise is caused by the increase in NEFA concentration since the plasma flow is largely unchanged during continuous exercise at the same intensity, emphasizing the important role of an increased adipose tissue NEFA release to enhance the plasma NEFA concentration driving the active muscle NEFA oxidation (Fig. 1 and Sect. 3) [8]. Consistent with these findings, an increase in the NEFA concentration via intralipid/heparin infusion increases the active muscle net NEFA uptake and fat oxidation [21], and even total fat oxidation at the relative high workload of 85 % of VO_2max_ [22]. Conversely, if NEFA concentrations are lowered via nicotinic acid infusion, the plasma NEFA oxidation is reduced [23]. The capacity for muscle to achieve such a rapid and manyfold increase in NEFA uptake upon contraction is remarkable, particularly in view of the manyfold increase in NEFA delivery caused by the massive increase in blood flow and the substantially reduced time available for interaction with the NEFA transporters. Of course, the 10- to 15-fold increase in blood flow with moderate-intensity exercise is accompanied by an increased capillary recruitment [19, 20], also referred to as nutritive flow [24], increasing the accessibility to NEFA transporters and diffusion surface. In addition, contraction-induced translocation of NEFA transporters from the intracellular storage pool to the cell membrane [11, 25] plays an important role in enhancing muscle NEFA uptake capacity during exercise. Another contributing factor is likely the high energy demand of the active muscle creating a NEFA surge and increasing the gradient for NEFA movement from the mitochondrion to the cytosol and eventual plasma NEFA uptake.

Over recent decades there has been considerable debate regarding whether NEFA transport across the plasma membrane occurs via passive diffusion or via facilitated transport by means of membrane-associated proteins. The physical properties of NEFA with a non-polar carbon chain and the polar head group would make passive diffusion possible, and this is seemingly supported by the above-described connection between NEFA concentration and active muscle NEFA uptake. However, over the past decade it has been shown that the majority of NEFA is transported across the cell membrane via protein-mediated mechanisms. Moreover, some of these fatty acid transporters demonstrate reversible translocation, from the intracellular storage pool to the cell membrane [11, 25] under the influence of contraction and insulin, analogous to the well-described glucose transporter type-4 (GLUT-4) translocation for muscle glucose uptake [7, 16]. Furthermore, studies with FAT/CD36 knockout or overexpressing mice [26, 27] have lent support to the conclusion that these fatty acid transporters critically regulate skeletal muscle fuel selection, performance, and training-induced adaptation of NEFA oxidation [26, 27]. However, while these studies undoubtedly demonstrate the importance of NEFA transporters in skeletal muscle NEFA transport from the plasma into the active muscle, they do not provide information on whether these transporters are rate limiting for NEFA oxidation. The well-described human in vivo increase in NEFA oxidation with exercise duration and the close linear relationship between NEFA delivery and uptake into the active muscle suggests that NEFA uptake is not solely controlled by the rate of sarcolemmal transport. This does not exclude an important regulatory role for fatty acid transporters and translocation of resting muscle NEFA uptake and its role in NEFA clearance from the circulation, particularly after food ingestion and during exercise in untrained individuals or patients with, for example, type 2 diabetes mellitus.

Once NEFA is taken up by the active muscle cell it can be oxidized or incorporated into intracellular lipid-like membranes and/or re-esterified and stored into IMTAG. The rate of resting muscle NEFA re-esterification into IMTAG is high [6, 28, 29], accounting for ~50 to 60 % of the total NEFA uptake [6, 30]. During exercise the majority (85–100 %) of NEFA taken up is directly oxidized [6, 10, 12–14, 17] and the rate of re-esterification into IMTAG is reduced, accounting for ~10 % of the total NEFA uptake [6]. A net IMTAG utilization with prolonged moderate-intensity exercise is observed for mixed muscle as a result of a reduced rate of IMTAG synthesis [6] and potentially an increased IMTAG lipolysis by adipose tissue TAG lipase responsible for the contraction-induced IMTAG lipolysis [31, 32]. However, in contrast to glycogen, the mixed muscle IMTAG store does not seem to be utilized to a large extent (20–50 % of the pre-exercise total IMTAG [5, 6, 33–35]), and this was even the case after 12 h of exercise [36]. The mixed muscle net IMTAG breakdown during exercise may underestimate IMTAG utilization by the different fiber types since only type I fibers have been suggested to utilize IMTAG during 2 h of 60 and 75 % of VO_2max_ bicycling exercise [37, 38] as well as after 2 h of high-intensity knee-extensor exercise [39]. Moreover, type II fibers have a much lower IMTAG content, but this does not decrease during exercise [37–39]. The high IMTAG synthesis rate that is reduced but still continues during muscle contraction together with a seemingly sub-maximal utilization rate suggests that the role of IMTAG is not solely as a rapidly responsive intracellular energy store such as glycogen, particularly since the higher circulatory NEFA oxidation shows that the NEFA oxidation capacity of mitochondria is not limiting for IMTAG utilization.

## Skeletal Muscle NEFA Supply from Adipose Tissue Fat Lipolysis During Exercise

Prolonged moderate-intensity exercise in the post-absorptive state causes a continuous increase in the NEFA concentration and oxidation with exercise duration [10, 17, 34, 40]. Moreover, the continuous increase in the NEFA concentration with duration of exercise implies that the rate of NEFA entering the circulation is higher than the uptake rate of the active muscles. Most of the circulatory NEFAs originate from TAG lipolysis in adipose tissue. Direct measurements across abdominal subcutaneous adipose tissue show a substantial increase in NEFA release at low exercise intensities [41]. Moreover, no or only a modest increase in adipose tissue NEFA release is found with higher exercise intensity [9, 41]. Therefore, low-intensity exercise provides an adequate stimulus for abdominal adipose tissue NEFA mobilization. The release of NEFA from subcutaneous adipose tissues depends on the relative contribution of three processes: (1) adipose tissue TAG lipolysis; (2) the rate of adipose tissue NEFA re-esterification into TAG; and (3) adipose tissue blood flow. The adipose tissue NEFA uptake is undetectable at rest [9, 42] and during exercise [9]; hence, NEFA re-esterification does not play an important role and the increase in adipose tissue NEFA release can be attributed to an increase in TAG lipolysis. However, the exercise-induced subcutaneous adipose tissue blood flow [9, 41, 43] and likely capillary recruitment [44, 45] plays a crucial role in the increase in NEFA release during exercise. The difference between the arterial and adipose tissue venous NEFA concentration is very similar at rest and during exercise. Therefore, the 2- to 3-fold increase in adipose tissue blood flow during exercise is responsible for the increase in the circulatory rate of NEFA [9]. The importance of adipose tissue blood flow for NEFA release is substantiated by observations in patients with type 2 diabetes who exhibit an exercise-stimulated adipose tissue lipolysis but in whom a high fraction of the liberated NEFA caused by TAG lipolysis could not be released into the circulation since the exercise-induced increase in adipose tissue blood flow was much smaller than in healthy subjects [46]. The exercise-induced increase in adipose tissue lipolysis and blood flow has mainly been attributed to the elevated catecholamine concentration with exercise [47, 48]. Moreover, circulating epinephrine is more important than sympathetic nervous activity for the stimulation of adipose tissue lipolysis during exercise [49, 50], albeit differences in various adipose tissue depots may exist [51]. Whereas epinephrine may be primarily responsible for the exercise-induced increase in lipolysis, selective β-adrenergic blockage does not completely prevent the increase in lipolysis [47, 48, 52], suggesting that other mediators play a role in the stimulation of lipolysis during exercise. Atrial natriuretic peptide is a potential candidate since there is evidence that it stimulates adipose tissue lipolysis and is produced during exercise in an intensity-dependent manner [53–55]. Growth hormone and cortisol increase adipose tissue lipolysis [56–58] and are potentially involved in the regulation of adipose tissue lipolysis during exercise. Growth hormone usually increases with exercise but its effect on lipolysis is not manifested until after 1–2 h of exercise [59] and thus is likely to become more important during prolonged exercise or be involved in the enhanced lipolysis during recovery from exercise [59]. Interestingly, 4 weeks of high-dose growth hormone supplementation raised the basal lipolytic rate substantially but did not change the exercise-induced increase in lipolysis during 30 min of exercise at 65 % of VO_2max_ [60], suggesting that growth hormone does not play an important role in the exercise-induced increase in adipose tissue lipolysis. Cortisol may increase to some extent with prolonged exercise [61] but this does not coincide with the rapid increase of adipose tissue TAG lipolysis upon the start of exercise. It has been suggested that infused cortisol and growth hormone stimulate systemic and adipose tissue lipolysis in an additive manner; however, it has to be mentioned that the cortisol levels were substantially higher than seen for any form of exercise [62]. Interleukin-6 (IL-6) is produced by skeletal muscle during exercise in increasing amounts with increased duration [63], and when recombinant human IL-6 (rhIL-6) is infused it increases systemic lipolysis [64]. Thus, IL-6 could be a signal from muscle to adipose tissue to coordinate the NEFA supply from adipose tissue with demand from the exercising muscle. However, concomitant IL-6 infusion and exercise fails to raise fatty acid delivery or oxidation [65], and at rest rhIL-6 infusion does not increase adipose NEFA release but does increase NEFA release from skeletal muscle [66]. Therefore, IL-6 is not a regulator for the increased adipose tissue NEFA release during exercise, but may be involved in skeletal muscle TAG utilization during exercise.

It should be noted that most research is performed in the post-absorptive state and little information exists on adipose tissue NEFA release during exercise in the post-prandial state. Ingestion of a mixed meal 1 h before exercise causes much lower plasma NEFA concentrations but does not influence adipose tissue lipolysis [67]. However, the adipose tissue net NEFA release is substantially lower during exercise in the post-prandial than in the fasted state as a result of the high adipose tissue NEFA uptake and re-esterification into TAG. In addition, the exercise-induced increase in adipose tissue blood flow is absent post-prandially [67]. Thus, the decrease in the muscle fat oxidation during exercise after a mixed meal may in part be caused by reduced NEFA delivery to the active muscle as a consequence of the decreased fatty acid mobilization from adipose tissue.

## Skeletal Muscle Fatty Acid Supply from Non-Adipose Tissue Fat Lipolysis During Exercise

Lipolysis of circulating TAG-rich lipoproteins in the capillaries of skeletal muscle is a source of NEFA for uptake and either oxidation or re-esterification in the active muscles. The majority of TAG-rich lipoproteins are the very-low-density lipoproteins (VLDL-TAG) produced in the liver in the fed and fasted state and contains one molecule of apolipoprotein B_100_ and 5–25,000 TAG molecules in its core [68]. In the fed state chylomicron TAG (CM-TAG) is secreted by the intestines and begins to appear in the blood approximately 1 h after the oral fat intake. The large CM-TAG contain apolipoprotein B_48_ and are very rich in TAG with a half-life estimated at 5 min [69]. Both VLDL-TAG and CM-TAG undergo lipolysis by lipoprotein lipase (LPL) anchored to heparin sulphate on the endothelial surfaces in muscle. The affinity of LPL for CM-TAG is reported to be 50-fold higher than for VLDL-TAG; however, this is balanced by the usually much higher concentration of VLDL-TAG. Moreover, complex differences exist between tissues in LPL expression, activity, and specific nutritional regulation [70, 71]. Circulating CM-TAGs are suggested to provide a more readily available source of NEFA for muscle oxidation than VLDL-TAG [72]. However, this obviously requires oral fat intake before or during the exercise, something that is not common practice among athletes. A key problem in the quantification of muscle CM-TAG and VLDL-TAG lipolysis is the technical difficulty of measuring TAG extraction across a muscle, a problem that is exaggerated during exercise when blood flow to the active muscle increases manyfold [73]. In general, no significant extraction of TAG, CM-TAG, and VLDL-TAG by the active or inactive muscle can be determined in the fasted state [10, 73, 74]. However, in spite of this inability to measure small quantities of muscle TAG extraction, the potential amount of NEFA for the muscle can contribute substantially to the total fat oxidation. Enevoldsen et al. [75] did not observe leg TAG uptake 1 h after meal ingestion, but during the following hour a significant TAG uptake of ~45 μmol/min was observed in only the exercising leg during 1 hour of cycle ergometer exercise at 50 % of VO_2max_. The NEFA uptake in the exercising leg was of similar magnitude; in other words, the NEFA uptake was only one-third of the TAG uptake in NEFA equivalents. Moreover, these results suggest that exercise per se increases muscle LPL activity, which has previously been shown not to occur via more direct muscle LPL activity measurements [76, 77]. However, Enevoldsen et al. [75] argued that not all capillaries are recruited in the resting muscle, but that the functional hyperemia of the active muscle increases the number of perfused capillaries [24, 78] and, hence, the accessible amount of LPL as depicted in Fig. 2. Evidence against a considerable contribution of circulating TAG to active muscle fat oxidation include findings that in the fasted state the muscle NEFA uptake and oxidation account for most of the fat oxidation [6, 10, 12, 14, 15, 17] and also for the majority of whole-body fat oxidation [37, 79], which suggests that relatively little of the oxidation of NEFAs originates from CM-TAG and VLDL-TAG. More research is needed to quantify the contribution of VLDL-TAG and CM-TAG to muscle fat oxidation and whether nutritional interventions before or during exercise can enhance its utilization without compromising performance.

## Discussion

In 1991, Weibel et al. [80] proposed the concept of symmorphosis. The concept states that animals and humans should be designed economically, i.e., that structural design should be matched to functional demand, or, in other words, no more structure is built and maintained than is required to meet functional demand. During very high-intensity exercise the concept of symmorphosis is put to the test as it is ultimately the active muscle that sets the aerobic demand because more than 90 % of energy is spent in the active muscle. The limitation of carbohydrate and lipid transfer from the microvascular system to the muscle cell seems to be reached at the onset of exercise and during continuous exercise above moderate intensities >50 % of VO_2max_. At higher workloads, intracellular stored substrates must be used for oxidation [1]. Glycogen can supply pyruvate up to the limit of maximal oxidation rates by the mitochondria, plus a further several-fold increase to fuel ‘anaerobic’ glycolysis. The question is, does IMTAG have a similar role to glycogen? The IMTAG droplets and the glycogen granules contain roughly equal amounts of energy and are in close vicinity to the mitochondria (Fig. 2). Indeed, IMTAG is utilized during moderate-intensity exercise, but in substantially lower quantities than plasma NEFA oxidation. In addition, when NEFA concentration/availability is low the IMTAG utilization is increased during moderate-intensity exercise, but it cannot compensate for the reduction in plasma NEFA oxidation [23, 81]. In other words, total fat oxidation by the active muscle is reduced. Enhanced muscle glycogen utilization has to cover the reduced NEFA oxidation when NEFA availability is low; if not, the ability to perform prolonged exercise is impaired [82]. It also demonstrates that NEFA uptake into the mitochondria and oxidation does not limit IMTAG utilization. Also different from glycogen is that IMTAG has an energy-consuming turnover rate of about 1–1.5 of the total IMTAG pool size/day, and, although IMTAG synthesis during exercise is reduced, it remains at a substantial rate [6] and it seems that the rate of complete IMTAG degradation to NEFA cannot be up-regulated very quickly in response to changes in demand. Therefore, it appears that IMTAG does not have the role of a rapidly responsive, directly available fat energy source without availability or transport limitations during exercise according to the concept of symmorphosis. Our knowledge of IMTAG turnover, lipolytic rate, and net utilization is almost completely lacking under conditions such as food intake and early versus late and moderate- versus high-intensity exercise, which makes any hypothesis on the role of IMTAG daunting. Perhaps IMTAG with its continuous high turnover rate may act as a ‘buffer’ for intramyocellular NEFAs, which in a high(er) concentration may be toxic [83, 84] and in a low(er) concentration compromises energy provision to the active muscles. The high synthesis and degradation rates for IMTAG mean it is able to fulfil this dual task. This role could be more important in slow twitch fibers with higher mitochondrial and IMTAG content and NEFA oxidation during exercise [37–39]. Research on fiber type differences and muscle NEFA content, IMTAG turnover, and NEFA transporters under various exercise conditions are needed to understand the role of IMTAG, particularly in various metabolic diseases.

Studies on patients with myophosphorylase deficiency or McArdle disease (also known as glycogen storage disease V) provide important insights into the role of circulatory glucose and NEFA versus intramuscular stored glycogen and IMTAG. Patients with McArdle disease cannot break down muscle glycogen and are characterized by a severely impaired exercise capacity, particularly during the initial 10 min of exercise, which is followed by sudden improvement in the exercise capacity referred to as the ‘second wind’ phenomenon. This second wind is attributed to an increase in blood glucose uptake [85] that is higher than in healthy individuals and is mediated by an increase in the number of GLUT-4 proteins or the localization in the membrane of GLUT-4 [86] and enhanced muscle blood flow [87]. Moreover, glucose supplementation greatly improves performance after ~5 min of exercise [88]. These findings underscore the important role of muscle glycogen to cover much of the instant manyfold increase in energy demand and the initial minute when the blood flow to the muscle is not yet fully increased to supply the necessary oxygen for substrate oxidation [18]. Moreover, the rate of glucose uptake by the active muscles is far lower than the glucose/glycogen substrate demand for high exercise intensities. During exercise, patients with McArdle disease oxidize more circulatory NEFA than healthy controls exercising at the same intensity [89], but enhanced availability via intralipid infusion does not improve performance [90]. The low rate of acetyl-coenzyme A (CoA) production from glycolysis cannot be replaced by a high rate of acetyl-CoA production from the β-oxidation likely related to reduced availability of oxaloacetate, tricarboxylic acid cycle intermediates, since ample pyruvate and/or amino acids are mandatory. Thus, glycogen is required to oxidize NEFA, whether from circulatory NEFA or IMTAG, with quantities the same as low to moderate intensities in healthy individuals required for higher exercise intensities in patients with McArdle disease.

Our understanding of fat metabolism during exercise is incomplete, not least the role of IMTAG. Apart from endurance training, most attempts to increase fat/NEFA oxidation to improve performance with the rationale of sparing the carbohydrates for high-intensity exercise have been rather unsuccessful. Endurance training increases the capacity of the muscle to take up NEFA [91], potentially by enhancing the number of capillaries and/or recruitment and the muscle NEFA oxidative capacity [91]. But how can we elevate the plasma NEFA concentration, which is an important determinant of the active muscle NEFA uptake and subsequent oxidation? Might there be an easier or additive way to enhance fat oxidation during exercise? This may be possible via ingestion of small quantities of fat before and/or during prolonged moderate-intensity exercise, which may aid the endogenous increase in NEFA release from adipose tissue, particularly during the initial 30 min of exercise when the NEFA concentration slightly decreases. However, oral glucose intake has been proven to enhance performance/endurance and should also be provided, but it will reduce the adipose tissue rate of lipolysis. Information is lacking but the simultaneous ingestion of starch with relatively small quantities of fat may potentially achieve the best results for both carbohydrate and fat utilization and, hence, performance.
